# Single local injection of 3S-HMGB1 enhances early bone healing and titanium implant osseointegration in type 2 diabetic mice

**DOI:** 10.1590/1678-7757-2025-0358

**Published:** 2025-10-08

**Authors:** Bhuvana Lakkasetter Chandrashekar, Alexandra Arteaga, Evelin Rios, Jimena Mora, Gustavo Pompermaier Garlet, Danieli C. Rodrigues, Claudia Cristina Biguetti

**Affiliations:** 1 The University of Texas at Dallas Erik Jonsson School of Engineering and Computer Science Department of Bioengineering Richardson Texas USA The University of Texas at Dallas, Erik Jonsson School of Engineering and Computer Science, Department of Bioengineering, Richardson, Texas, USA.; 2 The University of Texas Rio Grande Valley School of Podiatric Medicine Department of Podiatric Medicine Harlingen Texas USA The University of Texas Rio Grande Valley, Surgery and Biomechanics, School of Podiatric Medicine, Department of Podiatric Medicine, Harlingen, Texas, USA.; 3 Universidade de São Paulo Faculdade de Odontologia de Bauru Departamento de Ciências Biológicas Bauru SP Brasil Universidade de São Paulo, Faculdade de Odontologia de Bauru, Departamento de Ciências Biológicas, Bauru, SP, Brasil.

**Keywords:** Diabetes Mellitus, Oral Osseointegration, 3S-HMGB1, CXCR4

## Abstract

Diabetes significantly impairs bone healing via several mechanisms, including sustained inflammation, oxidative stress, and poor osteogenic responses. Fully reduced isoform of High Mobility Group Box 1 with three serine substitutions (3S-HMGB1) has shown regenerative properties while being resistant to oxidative degradation.

**Objective:**

This study evaluated the therapeutic potential of a single local injection of 3S-HMGB1 in early osseointegration under diabetic conditions in mice.

**Methodology:**

A total of 48 male 129/Sv mice (24 non-diabetic [ND], 24 diabetic [D]) received titanium implants following maxillary first molar extraction. ND and D mice (n:12) were injected with either saline (control) or 3S-HMGB1 (0.75 mg/kg) into the fresh extraction socket. Osseointegration was evaluated at 7 and 21 days post-implantation using microCT, histology (bone-to-implant contact [BIC] and birefringence), and immunohistochemistry for Runx2 and CXCR4.

**Results:**

ND controls exhibited early osteogenic activity, with a predominance of Runx2-positive cells at 7 days and successful osseointegration by 21 days. In contrast, D controls showed reduced numbers of Runx2-positive cells and markedly lower BV/TV, indicating compromised bone healing at 21 days. Treatment with 3S-HMGB1 resulted in significantly increased bone volume at the implant site in D animals (55.6±7.20% vs. 44.6±6.23%) and restored BIC from 44.9±9.32% (D controls) to 61.78±11.31% (D 3S-HMGB1), near ND levels (65.16±7.64%). Both ND and D groups treated with 3S-HMGB1 presented enhanced collagen organization. No significant differences were found in CXCR4 between groups; however, in D animals, a distinct peri-implant staining pattern suggested impaired recruitment despite preserved stem cell niches in the bone marrow.

**Conclusions:**

Collectively, our findings indicate that a single injection of redox-stable 3S-HMGB1 may represent a promising regenerative strategy to mitigate early implant failure in diabetes. Future studies should explore sustained delivery approaches to enhance long-term outcomes.

## Introduction

Systemic alterations in individuals with diabetes mellitus (DM) not only compromise metabolic balance but also affect cellular and molecular mechanisms essential for tissue regeneration, including immune regulation and stem cell recruitment.^1^ This significantly impacts implant treatment outcomes.^2–4^ Indeed, clinical and preclinical studies have consistently shown that individuals with DM are at a greater risk of impaired bone healing due to impaired tissue healing.^1,5–7^ Additionally, previous literature has shed light on how DM may be linked to the dysregulation of key cellular and molecular pathways involved in immune regulation, increased oxidative stress^1^, and deficiency in signaling molecules that rescue stem cell (SC) niches.^8^ For example, reduced expression of chemokines are used to attract cell progenitors, such as C-X-C motif chemokine ligand 12 (CXCL12; also known as stromal cell-derived factor 1 [SDF-1]).^9,10^ In bone healing, DM decreases the availability, proliferation capacity/recruitment of osteogenic and vascular progenitors,^11^ and reduces osteoblast differentiation.^8,12^ Similar changes have also been observed after immediate implant placement post tooth extraction in mice.^5^ Moreover, individuals with type 2 DM exhibit reduced frequencies of endothelial/pericyte progenitor cells (CD34+/CD146+ cells) in blood circulation, and reduced expression of C-X-C chemokine receptor type 4 (CXCR4) and CD34 receptors,^11^ which are important to support regenerative environments. CXCR4 is a chemokine receptor expressed in multiple cell types with migratory and/or repair function, including endothelial and hematopoietic stem cells (SCs), and skeletal mesenchymal SC (MSCs), and is the prototypic receptor for CXCL12.^13–15^ Thus, disruption of CXCL12/CXCR4 axis in diabetics decreases the chances for certain immune and progenitor stem cells to migrate towards repair sites.^14^

Amongst different cellular factors potentially involved in modulating inflammatory responses and osseointegration, High Mobility Group Box 1 (HMGB1) has been found to be essential in early osseointegration and tissue healing in mouse models.^16,17^ Its regenerative effects may, in part, be mediated through modulation of the CXCL12/CXCR4 axis, by forming a heterodimer with CXCL12 that enhances progenitor cell recruitment to the injury site.^17^ HMGB1 is the most abundantly expressed member of the HMG family, characterized by two DNA-binding domains (A and B boxes) that enable it to bend DNA and modulate chromatin structure, as well as gene expression.^18^ HMGB1 is among the most dynamic nuclear proteins, able to rapidly translocate to the different compartments of cytosol (1-2 seconds).^19^ This translocation may also include the active release of HMGB1 to the extracellular space in inflammatory environments.^20^ However, HMGB1 may also be passively released from necrotic cells in a fully reduced isoform as a damage associated molecular pattern (DAMP),^20,21^ which may be the case during surgical trauma following an implant placement.^16^ Interestingly, HMGB1 functional outcome depends heavily on its redox state and contextual signaling environment.^20^ This paradoxical role of HMGB1 is related to its spatial localization (intracellular and extracellular) and to its redox state, which determines the specific receptors that will be activated.^20^

HMGB1 exists in three redox states: fully reduced, disulfide, or oxidized. Fully reduced seems to be responsible for promoting tissue regeneration by forming a complex with CXCL12 and activating CXCR4;^17,22^ disulfide HMGB1 induces pro-inflammatory responses through TLR4 signaling;^17,20,22^ and oxidized HMGB1 is inactive and does not bind receptors.^22^ Among the three HMGB1 isoforms, the fully reduced form holds the greatest regenerative potential.^17,23,24^ However, since the diabetic environment is prone to overproducing reactive oxygen species (ROS), early oxidation of fully reduced HMGB1 is highly possible, rendering it either pro-inflammatory (disulfide isoform) or inactive (fully oxidized).^25,26^ To prevent HMGB1 oxidation, a synthetic fully reduced HMGB1 has been modified by replacing the three cysteine residues (C23, C45, and C106) with serines (3S-HMGB1),^22^ which remains in a reduced state. The isoform 3S-HMGB1 has been used to promote immunomodulation and tissue regeneration in various conditions,^17^ including tissue healing in murine diabetic models.^23,24^ Specifically, 3S-HMGB1 has been found to attract CXCR4+ skeletal SC by triggering the G_Alert_ state and increasing proliferation and early biomineralization in bone fractures of normoglycemic mice.^17^ Additionally, CXCR4/CXCL12/HMGB1 has been implicated in the recruitment of both inflammatory^27^ and regenerative cells during tissue healing.^14,17,28^ However, no studies to date have tested redox-stabilized HMGB1 in the context of implant osseointegration in diabetic individuals.

Thus, this study tested whether local delivery of 3S-HMGB1 enhances bone healing and implant osseointegration in a murine model of type 2 diabetes. Herein, we provide preclinical evidence using microtomographic, histological, and immunohistochemical analyses for Runt-Related Transcription Factor 2 (Runx2) and CXCR4. Understanding how this redox-stable regenerative form of 3S-HMGB1 influences osseointegration under diabetic conditions is essential for developing targeted therapeutic strategies to prevent early implant failure in this high-risk population.

## Methodology

### Study design

Animal experiments were performed according to ARRIVE guidelines.^29^ A total of 48 male wild type 129Sv mice, 6 weeks-old were purchased from Charles River Laboratories (Wilmington, MA, USA). Animals were maintained in specific pathogen-free conditions in the Vivarium at the University of Texas at Dallas (Richardson, TX) during all experimental phases. Experimental procedures were performed under supervision and approval from the Institutional Animal Care and Use Committee (IACUC #21-08). Animals were initially distributed into 2 groups, being 24 non-diabetic (ND) and 24 diabetic (D). Following surgery, 12 animals from each group (ND and D) were further distributed according to local injection treatment as: saline (Control) (*n*:12) or 3S-HMGB1 injection (*n*:12). Sample size was determined based on a prior study using the same immediate implant placement model in mice.^5^ Using a conservative effect size of Cohen's *d*=1.0 (two-sided α=0.05), *n*=6 per group provides >80% power; given prior observed effects (BV/TV *d* ≈ 1.54–3.48), power is expected to exceed 99% for this model. This shows that similar group sizes were sufficient to detect statistically significant differences in bone and histological outcomes between ND and D mice.

### Experimental protocol for diabetes induction and validation

This study employed a diabetic model using the 129Sv mouse strain and immediate implant placement post tooth extraction.^5^ Briefly, type 2 DM was induced by combination of high fat diet (HFD, Purina LabDiet5008 diet) and streptozotocin (STZ, S-0130, Sigma-Aldrich). HFD was introduced at the age of 6 weeks; when completing 10 weeks of age, animals were subjected to 2 spaced intraperitoneal (IP) injections of 100mg/Kg of STZ within an interval of 72 hours, as previously optimized.^30^ ND animals received 1x PBS as control. Fasting Plasma Glucose Levels (FPGL) were collected 7 days after the final STZ or 1xPBS injection to confirm diabetes status against control group (Supplementary Figure 1). Mice presenting FPGL from >250 mg/dL at day 7 after the last STZ dose were considered fully diabetic and were subjected to tooth extraction and implant placement.

### Dental implants and group treatments

Commercially pure titanium (cpTi, Grade 2) screws (0.50 mm ⌀ x 2 mm, Fairfax Dental Inc., Miami, FL, USA) were customized to perform the function of dental implants as previously described.^5^ Implants were cut to 1.5 mm in length, and the implant head was carefully polished to avoid cutting edges. Implant dimensions were confirmed using a caliper under stereomicroscope. Prior to surgery, screws were cleaned by sonication for 45 min each in acetone, DI water, and ethanol solutions, followed by sterilization at 121°C for 30 minutes. 3S-HMGB1, LPS-Free (REHM130 HMGB1) was acquired from HMGBiotech and reconstituted with sterile distilled water immediately prior to injection into implant sites.

### Immediate implant placement model

ND and D mice at the age of 11 weeks and weight ranging from 27-30 g were subjected to surgeries for tooth extraction followed by implant placement as previously described.^5^ Briefly, mice were anesthetized by inhalation of 4% isofluorane followed by an intramuscular (IM) injection of ketamine and xylazine (50-100 mg/kg; 20-50 mg/kg).^31^ The procedure involved extracting the maxillary right first upper molar from mice with minimal trauma using rat tooth forceps, followed by implant placement in the residual medial root alveolar socket, deemed adequate for implant space. A 0.45 mm pilot drill refined the implant bed. Prior to implant installation, the alveolar sockets of control ND an D groups received 10 μL of sterile 1xPBS using a microneedle, whereas the alveolar sockets of 3S-HMGB1 ND and D groups received 10 μL of 3S-HMGB1 (HMGBiotech) solution containing 0.75 mg/kg diluted in 1xPBS.^17^ Animals were randomly assigned from the diabetic and non-diabetic pools to receive either HMGB1 or 1×PBS treatments. Immediately after treatment, implants were installed in the residual alveolar socket of the palatine root using a micro-needle holder (Fine Science tools). All tooth extractions and implant placements were performed by a single experienced operator (CCB), ensuring consistency and eliminating inter-operator variability. Post-surgery, mice received 1.25 mg/kg buprenorphine SR subcutaneously for analgesia and were allowed free movement, water access, and a softened diet for 72 hours. Their health characteristics including feeding, drinking, grooming, and body weight were monitored daily. Mice were euthanized at 7- or 21-days post-implantation using sodium pentobarbital, and their maxillae were collected and preserved in 10% neutral-buffered formalin for further microCT and microscopic analysis.

### MicroCT

A total of six maxillae from each time point (7d and 21d), health status (ND and D), and alveolar socket treatment group (control vs. 3S-HMGB1) underwent microCT using ultra-high-resolution microCT imaging (OI/CT, MILabs, Utrecht, Netherlands). Samples were scanned as previously described, followed by reconstructing on MILabs (MILabs, Utrecht, Netherlands) at a voxel size of 20 μm. DICOM (Digital Imaging and Communications in Medicine) files were analyzed on Imalytics Preclinical (Gremse-IT GmbH, Aachen, Germany) for a total of 60 slides per animal. Volume of interest (VOI) was defined as the cylindrical region encompassing the peri-implant bone, extending 100 μm around the implant surface and matching the implant's full length within the bone, approximately 500μm.^31^ For the adjacent alveolar socket, VOI consisted of a cylinder with a 500 μm diameter confined within the socket boundaries, excluding the implant site. All measurements were performed using identical scanning parameters, reconstruction settings, and threshold values across samples to visualize and quantify total of bone volume (BV) surrounding the Ti implants, as well as bone volume fraction (BV/TV, %) in the alveolar socket of the first vestibular root.^5,16,31^

### Histological processing and analysis

After microCT scanning, specimens were washed to remove excess of 70% ethanol and subsequently immersed in 10% EDTA for two weeks for sample decalcification. After tissue processing, Ti screws were removed from their coronal portion using a microneedle holder. Semi-serial sections of 5 μm in thickness were obtained from the implant sites to be used for immunohistochemistry, birefringence, and Goldner Trichrome (GT) stain. Bone to implant contact (BIC%) was evaluated using GT stain as previously described.^5^ Qualitative patterns of connective tissue, bone deposition and osseointegration were evaluated using GT and picrosirius red following previous protocols.^5^ Briefly, BIC was measured using cellSens software (Olympus) by quantifying the length of alveolar bone in direct contact with the implant—within the defined implant space—relative to the total implant length at the bone interface (Supplementary Figure 2). BIC percentage was calculated as the proportion of bone contact over the entire implant length at bone level. Measurements were obtained after calibration, and two experienced histologists supervised the process and verified the BIC assessments performed by other evaluators. For picrosirius red, panoramic views of the implant sites were evaluated at 4x magnification with Ti spaces, and the interface of tissue surrounding the implant. Collagen organization was assessed under polarized light microscopy, where greenish birefringence indicates thin/immature (type 3) collagen fibers and red-orange birefringence denotes thick/mature (type I) collagen fibers.^31^ For all staining procedures, images were captured at the same magnification, and any brightness or contrast adjustments were applied uniformly across the entire image set to ensure consistency and comparability.

### Immunohistochemistry

Immunohistochemistry was used to identify and quantify markers related to CXCR4 (a receptor for 3S-HMGB1), and cells committed to osteoblastic differentiation (Runx2) in peri-implant tissues and surrounding bone. Briefly, histological slices were subjected to antigen retrieval using Citrate Buffer pH 6.0 at 95 °C for 30 min. Next, tissue samples were blocked with 1% Bovine serum albumin in 1x PBS (Sigma-Aldrich, St. Louis, MO, USA) for 30 minutes at room temperature. As primary antibodies, goat anti-mouse antibody CXCR4 (ThermoFisher # PA121625) at 1:100 and rabbit anti-mouse Runx2 at 1:500 (ab236639) were used. Tissue sections were incubated with each primary antibody at 4 °C overnight in a humidified chamber. Primary antibodies were subsequently incubated with goat or rabbit specific HRP/DAB (ABC) and Micropolymer Detection IHC Kit (Abcam, Cambridge, UK). At least three technical replicates from each sample were stained with each marker. A negative control was incubated with protein block instead of a primary antibody. After incubation, samples were washed, treated with hydrogen peroxide for 10 minutes to block endogenous activity, washed again in PBS (3x), and incubated using the Abcam IHC Micropolymer kit. Slides were developed with DAB chromogen for 1 minute and counterstained with Mayer's Hematoxylin for 2 minutes. Positive cells per field (400× magnification) were quantified using ImageJ software. For each animal, six representative fields were analyzed, with at least two technical replicates per sample to ensure consistency.^5^

### Statistical analysis

Statistical analysis of FPGL, MicroCT, BIC%, and immunohistochemistry data was performed using GraphPad Prism 10 (GraphPad Software Inc., San Diego, CA). Data distribution was assessed using the Shapiro–Wilk test. Non-parametric data were analyzed using the Kruskal–Wallis test, whereas normally distributed data were evaluated with one-way ANOVA. Multiple comparisons were made using post-hoc Tukey or Dunn's tests when applicable to evaluate significance between groups. Comparisons were made between time points within different groups (e.g., ND Control 7d vs. D Control 7d), between local treatments within same health status group (e.g., ND Control 7d vs. ND 3S-HMGB1 7d), and between same groups in different time points (e.g., ND Control 7d vs. ND Control 21d). A p-value ≤ 0.05 was considered statistically significant.

## Results

### Effective DM induction by HFD/STZ model

All ND animals had their FPGL measured 7 days after receiving cold 1×PBS injections (vehicle control for STZ). Glucose levels remained stable, with values of 93.50±18.50 mg/dL and 102.20±13.32 mg/dL. Animals were considered ND if their glucose levels remained below 250 mg/dL. D animals had their FPGL measured at two time points: 72 hours after the first STZ injection, and immediately prior to the second dose, with values averaging 114.80±36.65 mg/dL in the first measure for a batch of 24 animals. Seven days after the final intraperitoneal STZ injection, FPGL significantly increased to 301.00±53.99 mg/dL (p<0.0001). The value of one standard deviation below the mean was 278.2 mg/dL. No animals were discarded from the study (Supplementary Figure 1) and no implant losses were observed.

### MicroCT analysis reveals increased bone volume at the Ti implant interface in D animals

**Figure 1:**
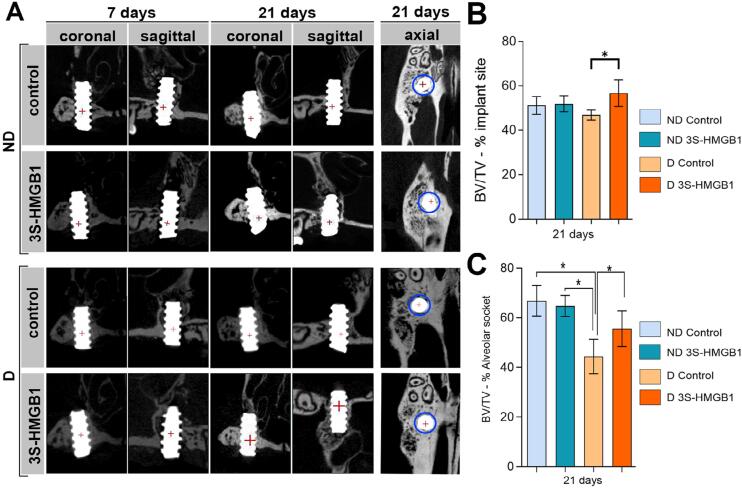
MicroCT analysis of alveolar sockets and implantation sites in 129Sv non-diabetic (ND) and diabetic (D) mice, control and 3S-HMGB1. A) 2D representative views (coronal, sagittal and axial) of the right hemimaxilla showing implant location in the palatine root socket at 7 and 21 days. Negligible amount of mineralized bone was observed at 7 days. B-C) Samples from 21 days were used for bone volume fraction (BV/TV, %) evaluation for implant sides (B) and alveolar sockets (C). Data represent mean±SD for n=6 animals per group (ND and D; control or 3S-HMGB1 treatment) analyzed at 21 days. Statistical analysis was performed using one-way ANOVA followed by Tukey's multiple comparison test (p < 0.05 considered significant).

MicroCT analysis revealed a significant increase in bone volume (BV/TV) at the implant site in diabetic animals treated with 3S-HMGB1 compared with controls (56.81±5.99% vs 46.95±2.25%, p < 0.05) at 21 days (Figure 1). In ND groups, 3S-HMGB1 did not significantly alter BV/TV values compared with ND controls (52.00±3.56% vs. 51.24±4.00%). In the alveolar socket, a similar trend was observed for BV/TV (%) in D groups, with D 3S-HMGB1 exhibiting a significant increase in BV/TV (55.6±7.20%) compared with D 21d controls (44.6±6.23%) (p<0.01). These results suggest that improved bone regeneration also occurred in the neighboring alveolar socket adjacent to the injection site, indicating that effects of 3S-HMGB1 extended beyond the implant interface. No significant differences were found between ND control and ND HMGB1 groups (66.85±6.18% vs. 64.78±4.26%).

### 3S-HMGB1 partially restores BIC in D conditions

**Figure 2:**
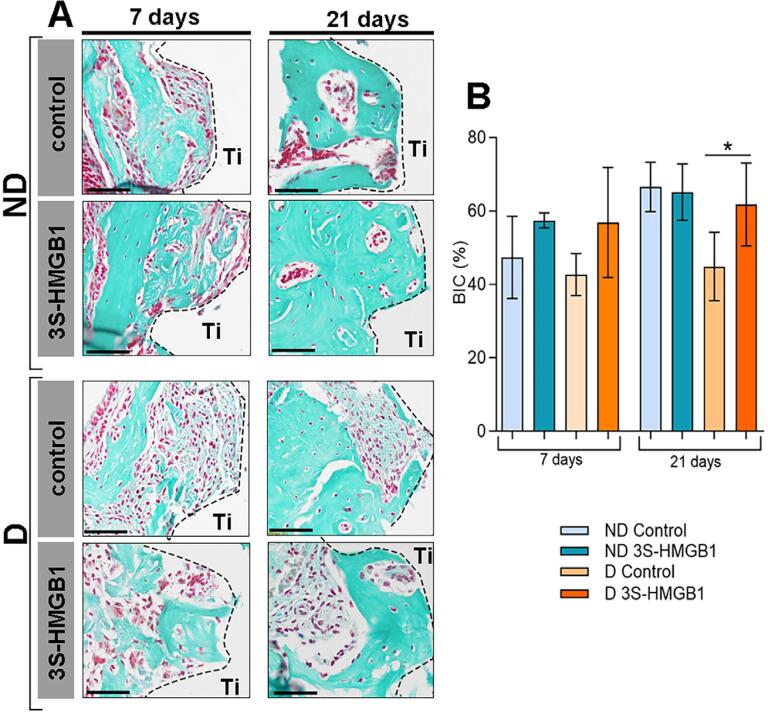
Goldner Thrichrome staining of peri-implant sites in non-diabetic (ND) and diabetic (D) mice at 7 and 21 days after implant placement. A) Representative images of the implant space previously occupied by titanium (Ti) screw is shown by "Ti"; Scale bar=100 μm. B) bone-to-implant contact (BIC) %. Data are presented as mean±SD for n=12 animals per group (ND and D; control or 3S-HMGB1 treatment), with 6 animals per time point (7 and 21 days). Statistical analysis was performed using one-way ANOVA followed by Tukey's multiple comparison test (p<0.05 considered significant).

Histomorphometry using Goldner's trichrome staining showed increased BIC in both ND and D groups treated with 3S-HMGB1 (Figure 2). At 7 days, BIC in D 3S-HMGB1 animals (56.9±15.0%) was notably higher than in D controls (42.67±5.73%) approaching levels seen in ND-treated animals (57.46±2.04%), however with no significant differences. At 21 days post-implant placement, BIC remained higher in D 3S-HMGB1 animals (61.78±11.31%) compared with D controls (44.9±9.32%) (p<0.05). ND groups showed no significant differences at this later time point (65.16±7.64% vs. 66.56±6.74%).

### Collagen organization and osteogenic marker expression

**Figure 3:**
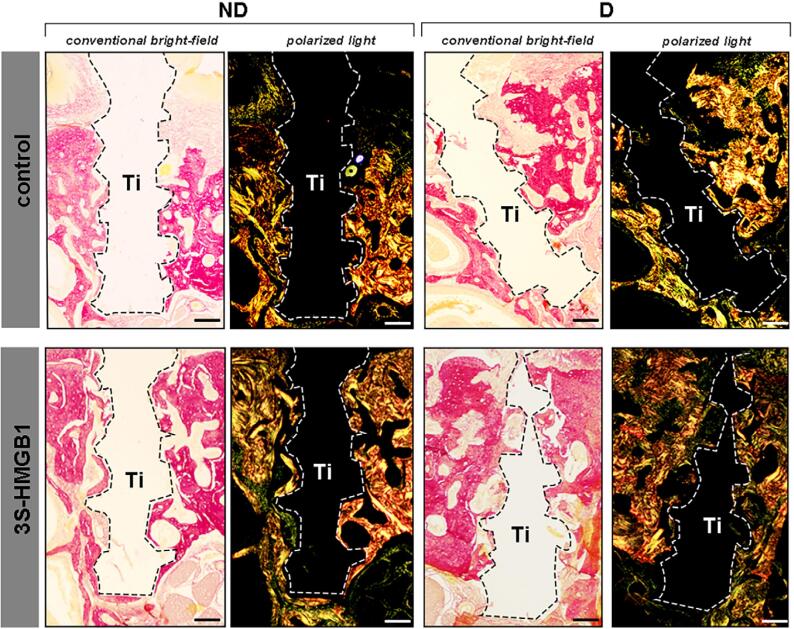
Birefringence evaluation of peri-implant sites in non-diabetic (ND) and diabetic (D) mice, treated or not with 3S-HMGB1, under conventional light and polarized light. Representative images are shown for 6 animals per group at 21 days post-implant placement (4× magnification). Implant space (Ti) is demonstrated between dashed lines. Staining: Sirius red. Scale bar 150 μm.

Picrosirius red staining revealed more organized and densely packed collagen fibers in both ND and D animals treated with 3S-HMGB1, with birefringent fibers ranging from orange to red and well demarcated implant threads, especially visible at 21 days. This contrasted with the less organized matrix seen in D control animals (Figure 3).

**Figure 4:**
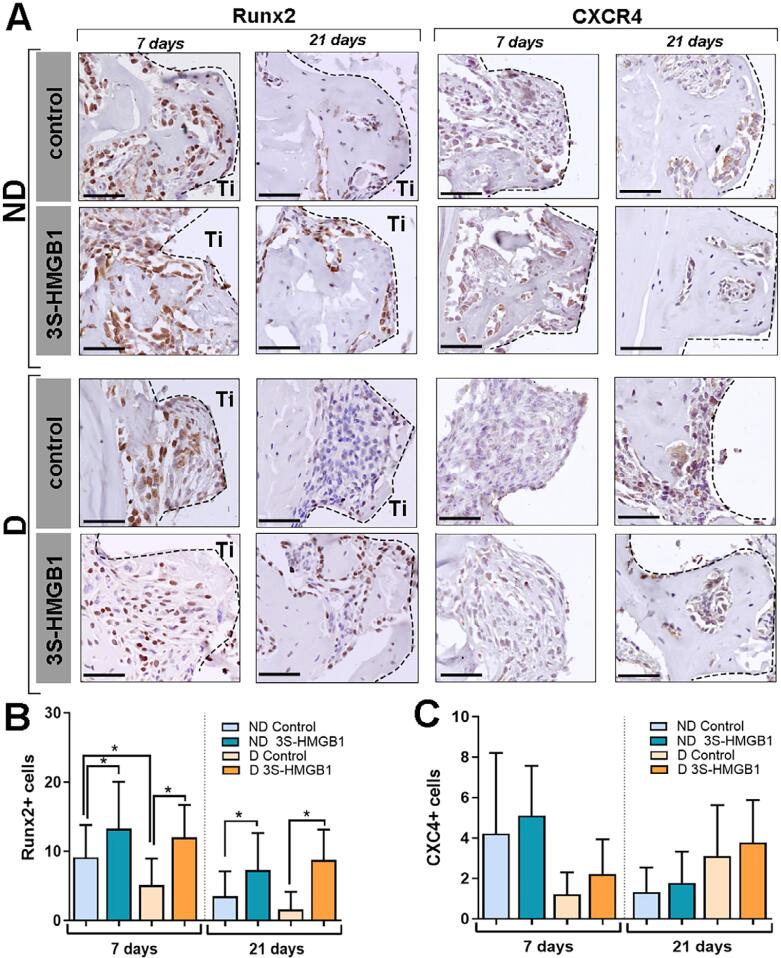
Immunohistochemistry for Runx2 and CXCR4 markers at the implant sites of non-diabetic (ND) and diabetic (D) mice, treated or not with 3S-HMGB1. A) Representative images from 6 animals per group at each time point are shown for each marker. Counterstaining Mayers hematoxylin, chromogen DAB. Scale bar 100 μm. B–C) Mean±SD of the number of positive cells per field (400× magnification). Data are presented as mean±SD for n=12 animals per group (ND and D; control or 3S-HMGB1 treatment), with 6 animals per time point (7 and 21 days). Statistical analysis was performed using one-way ANOVA followed by Tukey's multiple comparison test (p<0.05 considered significant).

To assess osteoblasts commitment, we evaluated Runx2 expression at the implant bone interface using immunohistochemistry (Figure 4A). Both ND and D 3S-HMGB1 treated groups showed strong signal across 7 and 21d time points. At 7 days, the number of Runx2 positive cells significantly increased in D 3S-HMGB1 animals (12.07±4.64%) compared with D controls (5.13±3.85%) (p<0.0001), with expression sustained at 21 days (8.79±4.37% vs. 1.65±2.51%) (p<0.0001). ND animals also showed significantly increased Runx2 expression with 3S-HMGB1 at both time points (p<0.05), though to a lesser extent compared with D groups (Figure 4B). These findings indicate that 3S-HMGB1 supports early osteoblast commitment and differentiation.

### CXCR4 expression reflects limited cell recruitment in diabetic animals

**Figure 5:**
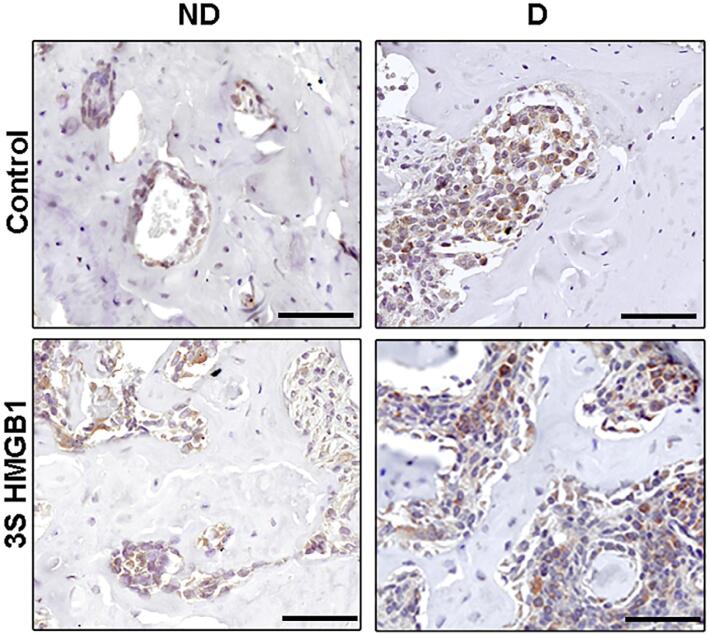
Representative images showing CXCR4 expression patterns at the implant sites and bone in non-diabetic (ND) and diabetic (D) mice at 21 days, treated or not with 3S-HMGB1. Counterstaining: Mayer's hematoxylin; chromogen: DAB indicating positive labeling, Scale bar 100 μm.

CXCR4 immunostaining showed low expression in peri-implant regions of D animals (Figure 4A), with only a modest increase following 3S-HMGB1 treatment (e.g., D 7d HMGB1: 2.22±1.72% vs. D 7d Control: 1.22±1.09%) (Figure 4B). No statistically significant difference was observed between each group. While CXCR4+ cells seem to be more abundant in ND animals, treatment modestly increased their numbers in both groups. Interestingly, bone marrow regions retained higher CXCR4 expression across all conditions (Figure 5), suggesting that stem cell niches remain intact in D animals, but chemotactic cues necessary for migration to the injury site may be impaired.

## Discussion

Previous studies in oral osseointegration have also shown DM-related adverse outcomes like poor bone healing and reduced osteogenic capacity are not solely due to systemic metabolic dysfunction, but also a result of local dysregulation from early bone healing responses.^4,5,12,32,33^ In this study, local administration of a redox-stable, non-oxidizable isoform of HMGB1 (3S-HMGB1) was used to bypass oxidative stresses found in diabetic inflammation and rescue the osseointegration potential in a diabetic mouse model.

This study employed a 129Sv mouse model of type 2 DM, induced by a HFD combined with low-dose STZ, to better replicate the advanced, decompensated hyperglycemic stages of the disease.^30,34^ The 129/Sv mouse strain was chosen as a preferred model for STZ-induced diabetes due to its consistent renal and gastric responses across various dosing regimens, offering an optimal balance between model robustness and animal welfare.^30^ As a validation of this model, D mice exhibited significantly elevated FPGL (301.00±53.99 mg/dL) prior to surgery and implant placement (Supplemental Figure 1). Following DM induction, mice were subjected to immediate implant placement post tooth extraction as previously validated.^5^ We observed that while ND controls exhibited satisfactory osteogenesis as early as 7 days, bone healing continued to progress by 21 days, resulting in satisfactory repair in both alveolar sockets and implant sites (Figure 1A–C). This was accompanied by improved collagen matrix organization (Figure 3) and increased bone-to-implant contact (BIC) (Figure 2B), consistent with previous findings in normoglycemic murine models.^5,31,35,36^ In contrast, D controls showed reduced bone formation and lower BIC at 21 days (Figure 2B), including alveolar sockets (Figure 1C), indicating impaired regenerative capacity under diabetic conditions, in agreement with previous findings.^5^ Moreover, D controls presented with a less organized collagen matrix compared with ND controls (Figure 3). Indeed, diabetic tissues are often marked by fragmented or disorganized collagen fibers, which contribute to mechanical weakness and impaired tissue remodeling.^4,37,38^ Previous evidence from experimental DM models also indicates impaired osseointegration associated with immature and disorganized newly bone formation.^4,34^

To evaluate the local effect of 3S-HMGB1 in implant integration, ND and D mice received either a single dose of 3S-HMGB1 or vehicle control injected into the fresh alveolar socket. This model was chosen as it represents a clinically feasible and practical approach for potential translation, involving a single injection of a regenerative molecule administered during surgery in clinical settings. ND and D animals treated with 3S-HMGB1 exhibited significantly increased bone volume formation surrounding not only the Ti implants (Figure 1B), but also within the remaining adjacent alveolar socket (Figure 1C). While no significant changes in BIC were noted between treated and untreated ND animals, 3S-HMGB1 treatment restored BIC values in diabetic animals to levels comparable to ND controls (Figure 2). Notably, qualitative analysis using picrosirius red staining revealed enhanced collagen matrix deposition in both ND and D groups at 21 days post-treatment, with 3S-HMGB1 mice presenting densely packed collagen fibers with organized birefringence and well-defined implant thread contours, ranging from orange to red under polarized light (Figure 3). The observation that collagen deposition in 3S-HMGB1-treated D animals resembled that of ND controls further suggests that this modified protein helps to restore the function of osteoblasts and the integrity of newly formed bone matrix. Comparable positive effects of 3S-HMGB1 have been described in fracture healing models in diabetic rodents, where different formulations of 3S or fully reduced HMGB1 were used with Ionic Liquid coatings.^23^ Other studies on fracture healing in normoglycemic mice, using a model of local injection and the same 3S-HMGB1 dosage as this study, have observed a boost in the local cell response, where 3S-HMGB1 provides enough cues for recruitment and differentiation of SCs.^17^

To further investigate the osteogenic effects of a single dose of 3S-HMGB1, we next examined osteoblast commitment. Osteoblast differentiation is tightly regulated by key signaling pathways, particularly the transcription factor Runx2 which drives the commitment and maturation of osteogenic precursors.^39^ In this study, strong Runx2 immunolabeling was observed at day 7 in both ND control and ND 3S-HMGB1 groups, as well as in D-3S-HMGB1 animals (Figure 4A). Runx2-positive cells presented significantly high numbers through day 21 in all groups treated with 3S-HMGB1, suggesting a sustained osteogenic response. In contrast, D-control animals exhibited a marked decline in Runx2-positive cells from day 7 to day 21, indicating impaired osteoblast commitment over time. These findings are consistent with previous studies reporting that 3S-HMGB1 enhances osteogenic differentiation by increasing ALP activity *in vitro* and promoting callus formation *in vivo*.^17^ Since ALP activity and other osteogenic markers are regulated by Runx2, the upregulation of this transcription factor in our study provides a mechanistic explanation for the improved bone healing observed. Conversely, under diabetic conditions, Runx2 expression is often significantly downregulated,^40^ contributing to impaired bone regeneration.^5^ Moreover, other recent work using hyperglycemic rodent models has shown that bone regeneration is markedly suppressed in such environments, with reduced expression of Runx2. Considered together, our results suggest that the therapeutic effects of 3S-HMGB1 may be mediated, at least in part, by the restoration of Runx2 expression and osteoblast function, even within the challenging diabetic microenvironment.

Since the primary role of 3S-HMGB1 is to attract CXCR4-expressing cells, we sought to investigate the distribution of cell types by morphological and spatial clues, and abundance of CXCR4-positive cells within the peri-implant region and nearby bone marrow (Figures 4 and 5). Interestingly, we observed no significant differences in the number of CXCR4+ cells surrounding Ti implants in treated versus untreated D animals. In D-control animals, CXCR4 expression occurred not only in mononuclear cells adjacent to implant threads but also within multinucleated osteoclasts, suggesting a broader range of cellular expression in response to hyperglycemia. The bone marrow of both ND and D animals—regardless of treatment—contained a relatively high abundance of CXCR4+ cells, indicating the preservation of stem cell niches even under D conditions within this model (Figure 5). However, the apparent lack of CXCR4+ cell recruitment to the peri-implant area in D animals suggests a possible defect in chemotactic signaling rather than cell availability. This is consistent with previous reports showing reduced expression of chemokines such as CXCL12 (the ligand for CXCR4) in the circulation of diabetic patients, which impairs the recruitment and retention of osteogenic and vascular progenitor cells.^11^ In the broader context of DM, multiple studies have reported diminished availability, proliferation, and migration of progenitor cell populations.^5,12^ These deficits are critical, as CXCR4 is broadly expressed in cells with migratory and reparative capacity, including M2 macrophages, lymphocytes, stromal fibroblasts, endothelial cells, hematopoietic stem cells, and skeletal mesenchymal stem cells (MSCs).^14,15,28^ In line with our findings, reduced recruitment of regenerative cells in diabetic animals may stem not from a lack of stem cells per se, but rather from an altered microenvironment that fails to generate adequate chemotactic cues. Thus, while 3S-HMGB1 may support tissue regeneration through CXCR4-mediated mechanisms, its efficacy could be limited by the broader dysregulation of the chemokine axis in diabetic conditions.

This study has some limitations that should be acknowledged. First, only male 129Sv mice were used. The rationale for this choice was to minimize variability in metabolic and hormonal profiles associated with the estrous cycle, which can influence bone turnover and tissue healing. While no sex-dependent differences in HMGB1 activity have been conclusively shown in the context of osseointegration, future work should address potential sex-related differences in treatment outcomes for this model. Second, our analyses were limited to short-term follow-up (7 and 21 days) and certain assays—such as CXCR4 expression—were evaluated at a single time point, potentially overlooking dynamic temporal changes. Indeed, the lack of significant differences at the earliest evaluation, particularly in diabetic animals, may reflect the time required for 3S-HMGB1 to exert its full biological activity following local delivery. Factors such as release kinetics, local stability, and tissue uptake are likely to influence the onset of its regenerative effects, and the *in vivo* pharmacokinetics of the redox-stable isoform in peri-implant tissues remain undetermined. Third, the findings are derived from a murine model of type 2 diabetes which, despite recapitulating key aspects of the human condition, does not fully replicate the complex pathophysiology of diabetic patients. Finally, we investigated only the local effects of a single 3S-HMGB1 injection; combining this approach with systemic diabetes management could potentially yield synergistic benefits for bone regeneration and osseointegration in diabetic settings. Thus, future studies should explore longer follow-up periods, additional time points, inclusion of both sexes, and the integration of metabolic control strategies to better inform clinical translation.

## Conclusion

Our findings indicate that a single local injection of 3S-HMGB1 at the time of immediate implant placement significantly improved early osseointegration by enhancing bone volume, collagen content and Runx2 expression in both normoglycemic and chronic hyperglycemic mice. While early healing effects were evident at 7 days, most pronounced improvements occurred at 21 days, reflecting advanced bone formation and maturation. These results provide important preclinical evidence supporting the potential of 3S-HMGB1 based therapies to reduce early implant failure in diabetic patients—a population at high risk for impaired bone healing. However, further studies are needed to assess long-term outcomes, dosage optimization, and mechanisms of action across different osseointegration stages. In this context, sustained- or controlled-release coating formulations could prolong 3S-HMGB1 local bioavailability, potentially amplifying regenerative effects and improving clinical translation. This remains a subject for future investigation and therefore requires exploration in future studies to enhance therapeutic potential in oral implantation.

## Data Availability

The supplementary materials generated during this study are available in the SciELO Data repository - https://doi.org/10.48331/SCIELODATA.SP5OX8

## References

[1] 1 Berbudi A, Rahmadika N, Tjahjadi AI, Ruslami R. Type 2 Diabetes and its impact on the immune system. Curr Diabetes Rev. 2020;16(5):442-9. doi: 10.2174/1573399815666191024085838 10.2174/1573399815666191024085838 PMC7475801 31657690

[2] 2 Wagner J, Spille JH, Wiltfang J, Naujokat H. Systematic review on diabetes mellitus and dental implants: an update. Int J Implant Dent. 2022 Jan 3;8(1):1. doi: 10.1186/s40729-021-00399-8 10.1186/s40729-021-00399-8 PMC8724342 34978649

[3] 3 Javed F, Romanos GE. Impact of diabetes mellitus and glycemic control on the osseointegration of dental implants: a systematic literature review. J Periodontol. 2009;80(11):1719-30. doi: 10.1902/jop.2009.090283 10.1902/jop.2009.090283 19905942

[4] 4 Nevins ML, Karimbux NY, Weber HP, Giannobile WV, Fiorellini JP. Wound healing around endosseous implants in experimental diabetes. Int J Oral Maxillofac Implants. 1998;3(5):620-9. 10.1016/S1079-2104(99)70236-2 9796145

[5] 5 Biguetti CC, Arteaga A, Chandrashekar BL, Rios E, Margolis R, Rodrigues DC. A Model of immediate implant placement to evaluate early osseointegration in 129/Sv diabetic mice. Int J Oral Maxillofac Implants. 2023;38(6):1200-10. doi: 10.11607/jomi.10335 10.11607/jomi.10335 PMC11181517 38085752

[6] 6 Chen Y, Zhou Y, Lin J, Zhang S. Challenges to improve bone healing under diabetic conditions. Front Endocrinol (Lausanne). 2022;13:861878. doi: 10.3389/fendo.2022.861878 10.3389/fendo.2022.861878 PMC8996179 35418946

[7] 7 Zhao YF, Zeng DL, Xia LG, Zhang SM, Xu LY, Jiang XQ, et al. Osteogenic potential of bone marrow stromal cells derived from streptozotocin-induced diabetic rats. Int J Mol Med. 2013;31(3):614-20. doi: 10.3892/ijmm.2013.1227 10.3892/ijmm.2013.1227 23292283

[8] 8 Tevlin R, Seo EY, Marecic O, McArdle A, Tong X, Zimdahl B, Malkovskiy A, et al. Pharmacological rescue of diabetic skeletal stem cell niches. Sci Transl Med. 2017;9(372):eaag2809. doi: 10.1126/scitranslmed.aag280 10.1126/scitranslmed.aag2809 PMC5725192 28077677

[9] 9 Kolluru GK, Bir SC, Kevil CG. Endothelial dysfunction and diabetes: effects on angiogenesis, vascular remodeling, and wound healing. Int J Vasc Med. 2012;2012:918267. doi: 10.1155/2012/918267 10.1155/2012/918267 PMC3348526 22611498

[10] 10 Gallagher KA, Liu ZJ, Xiao M, Chen H, Goldstein LJ, Buerk DG, et al. Diabetic impairments in NO-mediated endothelial progenitor cell mobilization and homing are reversed by hyperoxia and SDF-1 alpha. J Clin Invest. 2007;117(5):1249-59. doi: 10.1172/JCI29710 10.1172/JCI29710 PMC1857264 17476357

[11] 11 Terenzi DC, Al-Omran M, Quan A, Teoh H, Verma S, Hess DA. Circulating pro-vascular progenitor cell depletion during type 2 diabetes: translational insights into the prevention of ischemic complications in diabetes. JACC Basic Transl Sci. 2018;4(1):98-112. doi: 10.1016/j.jacbts.2018.10.005 10.1016/j.jacbts.2018.10.005 PMC6390504 30847424

[12] 12 Doherty L, Wan M, Kalajzic I, Sanjay A. Diabetes impairs periosteal progenitor regenerative potential. Bone. 2020;143:115764. doi: 10.1016/j.bone.2020.115764 10.1016/j.bone.2020.115764 PMC7770068 33221502

[13] 13 Lee G, Espirito Santo AI, Zwingenberger S, Cai L, Vogl T, Feldmann M, et al. Fully reduced HMGB1 accelerates the regeneration of multiple tissues by transitioning stem cells to GAlert. Proc Natl Acad Sci U S A. 2018;115(19):E4463-72. doi: 10.1073/pnas.1802893115 10.1073/pnas.1802893115 PMC5949009 29674451

[14] 14 Haque N, Fareez IM, Fong LF, Mandal C, Abu Kasim NH, Kacharaju KR, et al. Role of the CXCR4-SDF1-HMGB1 pathway in the directional migration of cells and regeneration of affected organs. World J Stem Cells. 2020;12(9):938-51. doi: 10.4252/wjsc.v12.i9.938 10.4252/wjsc.v12.i9.938 PMC7524697 33033556

[15] 15 Pozzobon T, Goldoni G, Viola A, Molon B. CXCR4 signaling in health and disease. Immunol Lett. 2016;177:6-15. doi: 10.1016/j.imlet.2016.06.006 10.1016/j.imlet.2016.06.006 27363619

[16] 16 Biguetti CC, Cavalla F, Silveira EV, Tabanez AP, Francisconi CF, Taga R, et al. HGMB1 and RAGE as essential components of Ti osseointegration process in mice. Front Immunol. 2019;10:709. doi: 10.3389/fimmu.2019.00709 10.3389/fimmu.2019.00709 PMC6461067 31024546

[17] 17 Lee G, Espirito Santo AI, Zwingenberger S, Cai L, Vogl T, Feldmann M, et al. Fully reduced HMGB1 accelerates the regeneration of multiple tissues by transitioning stem cells to GAlert. Proc Natl Acad Sci U S A. 2018;115(19):E4463-72. doi: 10.1073/pnas.1802893115 10.1073/pnas.1802893115 PMC5949009 29674451

[18] 18 Tang D, Kang R, Zeh HJ, Lotze MT. The multifunctional protein HMGB1: 50 years of discovery. Nat Rev Immunol. 2023;23(12):824-41. doi: 10.1038/s41577-023-00894-6 10.1038/s41577-023-00894-6 37322174

[19] 19 SSapojnikova N, Maman J, Myers FA, Thorne AW, Vorobyev VI, Crane-Robinson C. Biochemical observation of the rapid mobility of nuclear HMGB1. Biochim Biophys Acta. 2005;1729(1):57-63. doi: 10.1016/j.bbaexp.2005.03.002 10.1016/j.bbaexp.2005.03.002 15823506

[20] 20 Tang D, Billiar TR, Lotze MT. A Janus tale of two active high mobility group box 1 (HMGB1) redox states. Mol Med. 2012;18(1):1360-2. doi: 10.2119/molmed.2012.00314 10.2119/molmed.2012.00314 PMC3533642 23073660

[21] 21 Kang R, Chen R, Zhang Q, Hou W, Wu S, Cao L, et al. HMGB1 in health and disease. Mol Aspects Med. 2014;40:1-116. doi: 10.1016/j.mam.2014.05.001 10.1016/j.mam.2014.05.001 PMC4254084 25010388

[22] 22 Venereau E, Casalgrandi M, Schiraldi M, Antoine DJ, Cattaneo A, De Marchis F, et al Mutually exclusive redox forms of HMGB1 promote cell recruitment or proinflammatory cytokine release. J Exp Med. 2012;209(9):1519-28. doi: 10.1084/jem.20120189 10.1084/jem.20120189 PMC3428943 22869893

[23] 23 Arteaga A, Biguetti CC, Chandrashekar B, La Fontaine J, Rodrigues DC. Revolutionizing fracture fixation in diabetic and non-diabetic rats: High mobility group box 1-based coating for enhanced osseointegration. Bone. 2023;177:116917. doi: 10.1016/j.bone.2023.116917 10.1016/j.bone.2023.116917 PMC11292581 37739297

[24] 24 Biscetti F, Straface G, De Cristofaro R, Lancellotti S, Rizzo P, Arena V, et al. High-mobility group box-1 protein promotes angiogenesis after peripheral ischemia in diabetic mice through a VEGF-dependent mechanism. Diabetes. 2010;59(6):1496-505. doi: 10.2337/db09-1507 10.2337/db09-1507 PMC2874711 20200317

[25] 25 Ferrara M, Chialli G, Ferreira LM, Ruggieri E, Careccia G, Preti A, et al. Oxidation of HMGB1 is a dynamically regulated process in physiological and pathological conditions. Front Immunol. 2020;11:1122. doi: 10.3389/fimmu.2020.01122 10.3389/fimmu.2020.01122 PMC7326777 32670275

[26] 26 Vénéreau E, Ceriotti C, Bianchi ME. DAMPs from cell death to new life. Front Immunol. 2015;6:422. doi: 10.3389/fimmu.2015.00422 10.3389/fimmu.2015.00422 PMC4539554 26347745

[27] 27 Schiraldi M, Raucci A, Muñoz LM, Livoti E, Celona B, Venereau E, et al. HMGB1 promotes recruitment of inflammatory cells to damaged tissues by forming a complex with CXCL12 and signaling via CXCR4. J Exp Med. 2012;209(3):551-63. doi: 10.1084/jem.20111739 10.1084/jem.20111739 PMC3302219 22370717

[28] 28 Di Maggio S, Milano G, De Marchis F, D’Ambrosio A, Bertolotti M, Palacios BS, et al. Non-oxidizable HMGB1 induces cardiac fibroblasts migration via CXCR4 in a CXCL12-independent manner and worsens tissue remodeling after myocardial infarction. Biochim Biophys Acta Mol Basis Dis. 2017;1863(11):2693-704. doi: 10.1016/j.bbadis.2017.07.012 10.1016/j.bbadis.2017.07.012 28716707

[29] 29 Kilkenny C, Browne W, Cuthill IC, Emerson M, Altman DG; NC3Rs Reporting Guidelines Working Group. Animal research: reporting *in vivo* experiments: the ARRIVE guidelines. Br J Pharmacol. 2010;160(7):1577-9. doi: 10.1111/j.1476-5381.2010.00872.x 10.1111/j.1476-5381.2010.00872.x PMC2936830 20649561

[30] 30 Nørgaard SA, Søndergaard H, Sørensen DB, Galsgaard ED, Hess C, Sand FW. Optimising streptozotocin dosing to minimise renal toxicity and impairment of stomach emptying in male 129/Sv mice. Lab Anim. 2020 Aug 1;54(4):341–52. 10.1177/0023677219872224 31510860

[31] 31 Biguetti CC, Cavalla F, Silveira EM, Fonseca AC, Vieira AE, Tabanez AP, et al. Oral implant osseointegration model in C57Bl/6 mice: microtomographic, histological, histomorphometric and molecular characterization. J Appl Oral Sci. 2018;26:e20170601. doi: 10.1590/1678-7757-2017-0601 10.1590/1678-7757-2017-0601 PMC5963915 29898187

[32] 32 Chrcanovic BR, Albrektsson T, Wennerberg A. Diabetes and oral implant failure: a systematic review. J Dent Res. 2014;93(9):859-67. doi: 10.1177/0022034514538820 10.1177/0022034514538820 PMC4541101 24928096

[33] 33 Hasegawa H, Ozawa S, Hashimoto K, Takeichi T, Ogawa T. Type 2 diabetes impairs implant osseointegration capacity in rats. Int J Oral Maxillofac Implants. 2008;23(2):237-46. 18548919

[34] 34 King S, Klineberg I, Levinger I, Brennan-Speranza TC. The effect of hyperglycaemia on osseointegration: a review of animal models of diabetes mellitus and titanium implant placement. Arch Osteoporos. 2016;11(1):29. doi: 10.1007/s11657-016-0284-1 10.1007/s11657-016-0284-1 27637755

[35] 35 Wheelis SE, Biguetti CC, Natarajan S, Arteaga A, El Allami J, Lakkasettar Chandrashekar B, et al. Cellular and molecular dynamics during early oral osseointegration: a comprehensive characterization in the Lewis rat. ACS Biomater Sci Eng. 2021;7(6):2392-407. doi: 10.1021/acsbiomaterials.0c01420 10.1021/acsbiomaterials.0c01420 PMC8796703 33625829

[36] 36 Mouraret S, Hunter DJ, Bardet C, Brunski JB, Bouchard P, Helms JA. A pre-clinical murine model of oral implant osseointegration. Bone. 2014;58:177-84. doi: 10.1016/j.bone.2013.07.021 10.1016/j.bone.2013.07.021 PMC4962868 23886841

[37] 37 Wajima CS, Pitol-Palin L, Batista FR, Santos PH, Matsushita DH, Okamoto R. Morphological and biomechanical characterization of long bones and peri-implant bone repair in type 2 diabetic rats treated with resveratrol. Sci Rep. 2024;14(1):2860. doi: 10.1038/s41598-024-53260-4 10.1038/s41598-024-53260-4 PMC10838324 38310154

[38] 38 Pitol-Palin L, Batista FR, Gomes-Ferreira PH, Mulinari-Santos G, Ervolino E, Souza FA, et al. Different stages of alveolar bone repair process are compromised in the type 2 diabetes condition: an experimental study in rats. Biology (Basel). 2020;9(12):471. doi: 10.3390/biology9120471 10.3390/biology9120471 PMC7766949 33339217

[39] 39 Phimphilai M, Zhao Z, Boules H, Roca H, Franceschi RT. BMP signaling is required for RUNX2-dependent induction of the osteoblast phenotype. J Bone Miner Res. 2006;21(4):637-46. doi: 10.1359/jbmr.060109 10.1359/JBMR.060109 PMC2435171 16598384

[40] 40 Fowlkes JL, Bunn RC, Liu L, Wahl EC, Coleman HN, Cockrell GE, et al. Runt-related transcription factor 2 (RUNX2) and RUNX2-related osteogenic genes are down-regulated throughout osteogenesis in type 1 diabetes mellitus. Endocrinology. 2008;149(4):1697-704. doi: 10.1210/en.2007-1408 10.1210/en.2007-1408 PMC2276714 18162513

